# Fabrication of 2-Inch Free-Standing GaN Substrate on Sapphire With a Combined Buffer Layer by HVPE

**DOI:** 10.3389/fchem.2021.671720

**Published:** 2021-04-22

**Authors:** Nanliu Liu, Yongjing Jiang, Jian Xiao, Zhiwen Liang, Qi Wang, Guoyi Zhang

**Affiliations:** ^1^School of Physics and Electronics, Qiannan Normal University for Nationalities, Duyun, China; ^2^Dongguan Institute of Opto-Electronics, Peking University, Dongguan, China; ^3^Research Center for Wide-Gap Semiconductors, State Key Laboratory for Artificial Microstructures and Mesoscopic Physics, School of Physics, Peking University, Beijing, China

**Keywords:** free-standing GaN substrate, LT-AlN, pre-annealing, combined buffer layer, 3D GaN interlayer, HVPE

## Abstract

Free-standing GaN substrates are urgently needed to fabricate high-power GaN-based devices. In this study, 2-inch free-standing GaN substrates with a thickness of ~250 μm were successfully fabricated on double-polished sapphire substrates, by taking advantage of a combined buffer layer using hydride vapor phase epitaxy (HVPE) and the laser lift-off technique. Such combined buffer layer intentionally introduced a thin AlN layer, using a mix of physical and chemical vapor deposition at a relatively low temperature, a 3-dimensional GaN interlayer grown under excess ambient H_2_, and a coalescent GaN layer. It was found that the cracks in the epitaxial GaN layer could be effectively suppressed due to the large size and orderly orientation of the AlN nucleus caused by pre-annealing treatment. With the addition of a 3D GaN interlayer, the crystal quality of the GaN epitaxial films was further improved. The 250-μm thick GaN film showed an improved crystalline quality. The full width at half-maximums for GaN (002) and GaN (102), respectively dropped from 245 and 412 to 123 and 151 arcsec, relative to those without the 3D GaN interlayer. The underlying mechanisms for the improvement of crystal quality were assessed. This method may provide a practical route for fabricating free-standing GaN substrates at low cost with HVPE.

## Introduction

GaN-based devices have experienced important development for applications in light-emitting diodes, radio frequency devices, and electronics (Sandvik et al., 2001; Chai et al., 2018; Li et al., 2018). However, these devices are generally constructed on foreign substrates, such as SiC, Si, and patterned sapphire substrates (PSSs). For short-wavelength laser diodes and for high-power and high-frequency devices, native GaN substrates have many advantages, such as their low level of current leakage and long lifetimes due to the high quality of the active epitaxial layer, accompanied by low dislocation densities and a lower level of lattice distortion, derived from homo-epitaxy (Liu et al., 2017; Sumiya et al., 2017; Han et al., 2018).

Generally speaking, hydride vapor-phase epitaxy (HVPE) is the most commonly used technique to obtain free-standing GaN substrates, due to the high growth rate it produces and its low cost (Fujito et al., 2009). Normally, the procedure for fabricating free-standing GaN substrates includes the following steps. First, a GaN template with a low temperature GaN buffer layer (LT-GaN), grown with metal organic chemical vapor deposition (MOCVD) is used for the re-growth of GaN film in HVPE. Following that, a GaN film, with a thickness of several 100 μm, is then separated from its foreign substrate using self-separation (Lee et al., 2009) or the laser lift-off (LLO) technique (Paskova et al., 2006). However, the residual strain and lattice distortion caused by hetero-epitaxy could not be completely suppressed using the LT-GaN buffer layer method. It has recently been reported that the crystal quality of GaN film and related devices were improved when the LT-GaN buffer layer was replaced with a thin AlN buffer layer on both PSS and plain sapphire substrate, due to the improvement of the crystal quality of the GaN nucleation layer (Chen et al., 2015, 2016). The AlN buffer layer can also be used in a cost-effective way because it can be deposited either through a sputter or with physical vapor deposition (PVD), using cheaper appliances than MOCVD.

In this study, GaN thick film was directly grown on a double-polished sapphire substrate with a LT-AlN buffer layer using HVPE. The GaN thick films grown on a pre-annealed LT-AlN layer and a 3-dimensional (3D) GaN interlayer grown under the excess ambient H_2_, the variation in the morphology and crystal quality of LT-AlN and GaN films were studied with differential-interference contrast microscopy (DICM), atomic-force microscopy (AFM), and X-ray diffraction (XRD). The behaviors of GaN nucleation were also investigated, and they further testify to the effects of pre-annealing the LT-AlN layer. Two-inch free-standing GaN substrate with a high crystal quality was obtained with LLO.

## Materials and Methods

**Figure 1:**
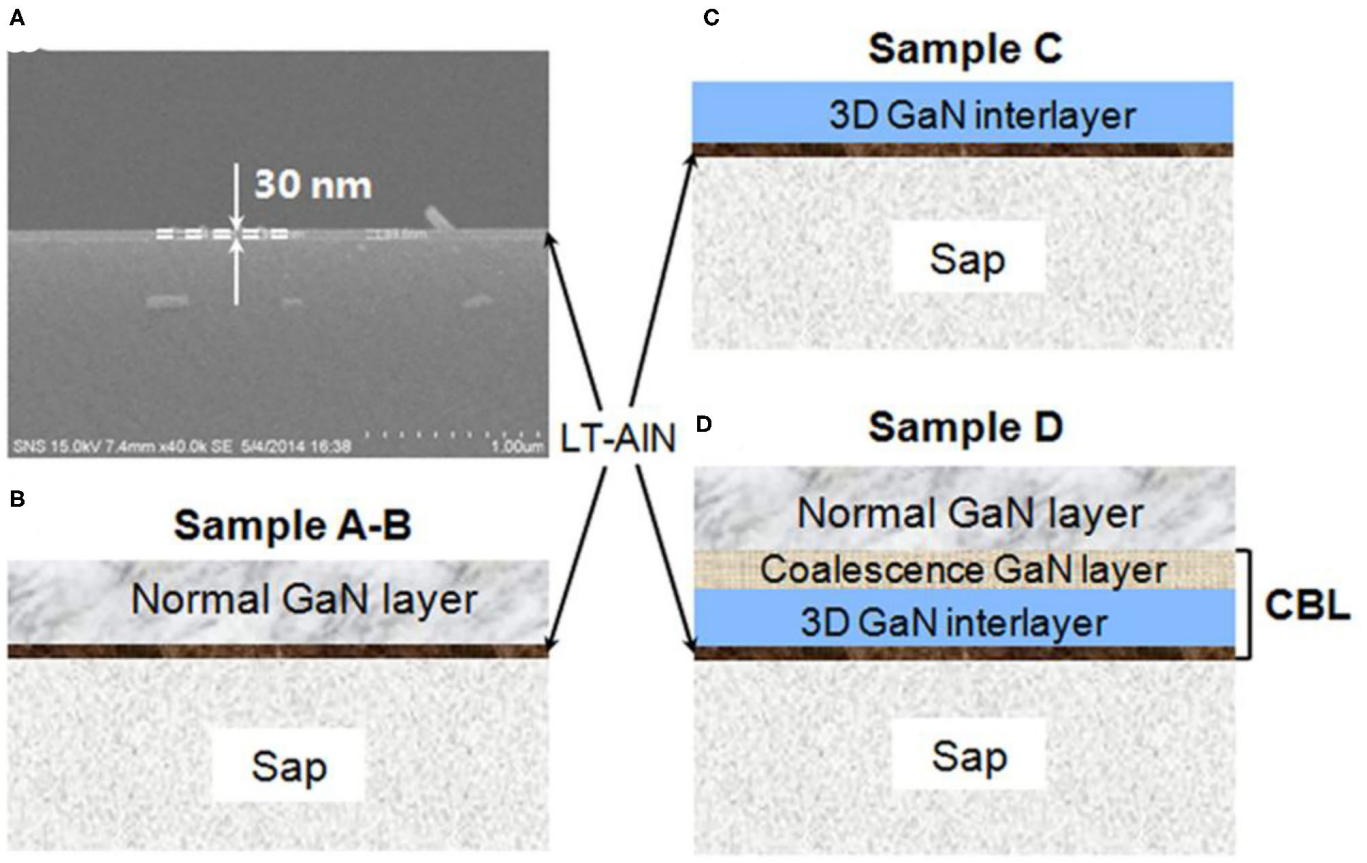
**(A)** SEM image of a cross-section of a double-polished sapphire covered with LT-AlN. **(B–D)** Schematic diagrams of the processes designed for GaN growth.

A thin AlN buffer layer (labeled LT-AlN) with a thickness of 30 nm was deposited on the surface of commercial double-polished sapphire substrates at a temperature below 200°C using a mix of physical and chemical vapor deposition system (Liu et al., 2018). The LT-AlN can be clearly observed in the SEM image in Figure 1A. All of the sapphire substrates that were covered with LT-AlN in the experiment had previously been cleaned and dried using a routine process (Liu et al., 2016).

First, to investigate the effects of the annealing process of LT-AlN on the growth of the GaN film on top of it, comparative studies were performed under normal growth conditions (NGCs) using HVPE, as shown in Figure 1B. Samples that did not undergo pre-annealing treatment were called sample A. Sample B samples were annealed under mixed gases N_2_/NH_3_ gases at 3/1 and 1070°C for 8 min prior to the GaN epitaxial growth. Then, samples A and B were loaded into our home-made HVPE reactor for thick GaN film growth. During the NGC processing, the carrier gas was a mixture of N_2_ and H_2_ (N_2_/H_2_ = 1/1). The growth pressure and the V/III ratio of source gases was set to 550 torr and 800/20, respectively. Under these conditions, a thick GaN layer, with thicknesses ranging from 20 to 250 μm, was grown on the LT-AlN at 1070°C.

A modified structure with a 3D GaN interlayer was then proposed, as shown in Figure 1C. In this structure, samples (labeled sample C) were first annealed under the same conditions as sample B, and then extra H_2_ gas at a flow rate of 10 slm was added into the growth zone during HVPE growth. Here, the V/III ratio of source gases was set to 100/1 for the 3D GaN interlayer growth for about 1000 s. For sample D, shown in Figure 1D, the V/III ratio of source gases was changed to 60/1 to initiate the coalescence growth of the GaN layer for another 1000 s (Zhao et al., 2007) after the series of processes for sample C were completed. Then, a normal GaN layer was then programmed for growth for 1–3 h.

The surface and cross-sectional morphologies of the samples were characterized with DICM, AFM, and scanning electron microscope (SEM). The DICM was implemented with a Leica DM 2700 RL, and the AFM tapping mode was performed in Bruker Dimension ICON. The SEM images were obtained using a field-emission scanning electron microscope (Zeiss, SIGMA 300), and energy dispersive spectroscopy (EDS) testing was conducted with a Bruker Quantax 400 system. High-resolution XRD was measured using a Bruker D8 Discovery system.

## Results and Discussion

### Effects of Pre-annealing of LT-AlN on the Crystalline Quality of GaN Film

**Figure 2:**
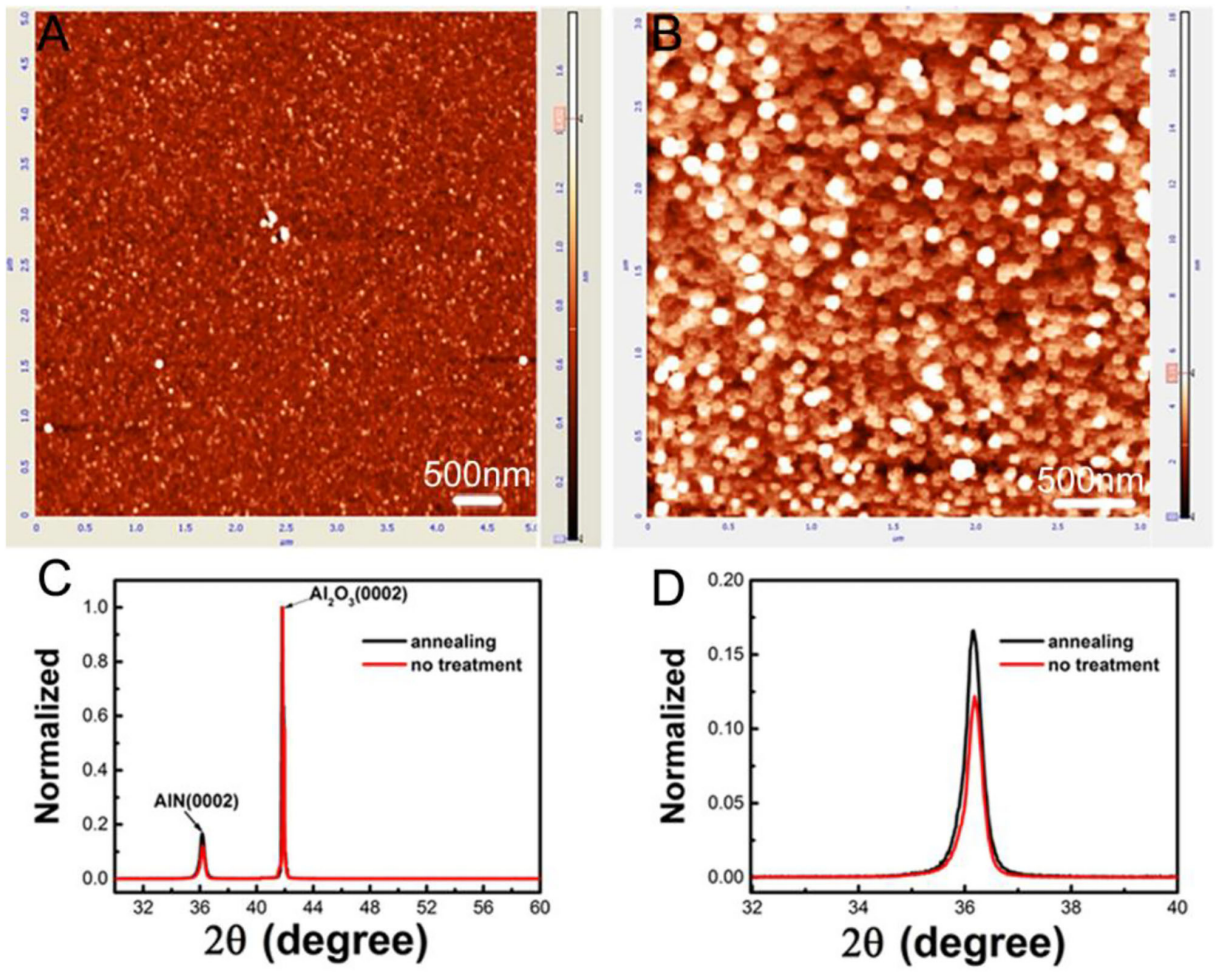
AFM images of LT-AlN without **(A)** and with **(B)** annealing treatment. **(C)** ω-2θ scan patterns of the (002) plane peak of LT-AlN with and without annealing treatment. **(D)** Magnification of **(C)**.

Figure 2 shows the AFM images and the XRD measurements for LT-AlN with and without annealing treatment. As shown in Figures 2A,B, after annealing treatment, the surface roughness of the AlN film increased from 0.27 to 1.20 nm, and the nucleus of AlN exhibited an obvious hexagon columnar structure, indicating a highly oriented and increased crystal quality for the AlN nucleus. In addition, the size of the nucleus of AlN was larger than it was before annealing treatment.

The results of the ω-2θ scan patterns for the (002) peak of LT-AlN with high-resolution XRD characterization also confirmed the improvement of the crystal quality of the AlN nucleus after the annealing process, as shown in Figures 2C,D. After the annealing treatment, the intensity of the (002) peak of LT-AlN film showed an obvious increase, which indicates an improvement for the AlN crystal quality, mainly due to obvious grain coarsening and orientation following annealing, as shown in Figure 2B (Okuno et al., 2013).

**Figure 3:**
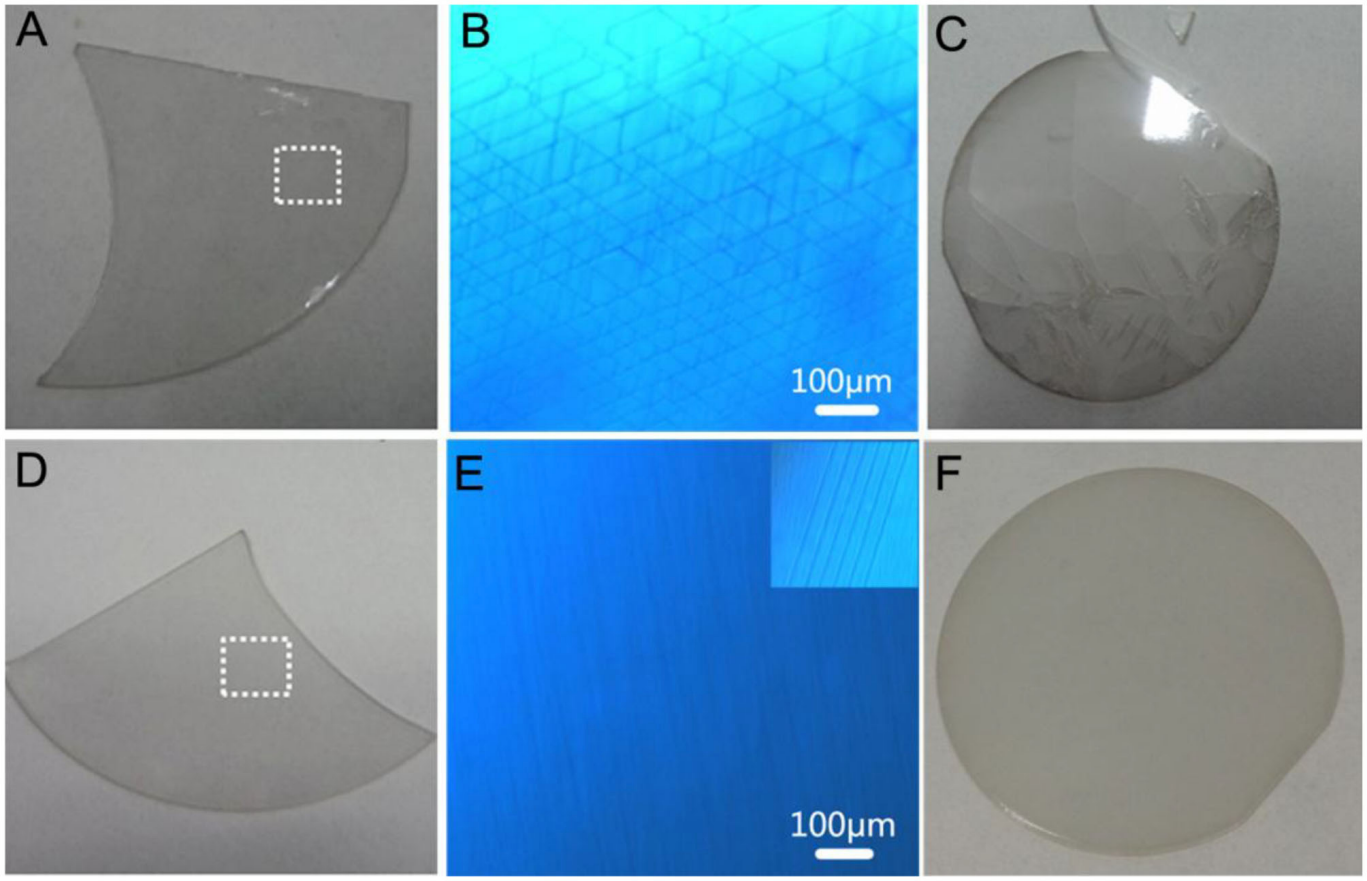
Photographic and optical microscopic images of GaN films grown above LT-AlN with no annealing treatment **(A–C)** (sample A) and with annealing pre-treatment **(D–F)** (sample B) [the thicknesses of GaN for **(A,D,C,F)** are 20 and 100 μm, respectively]. **(B,E)** Microscopic images of the dot white rectangle of **(A,D)**, respectively, where the insert of **(E)** shows the local micro-crack in **(D)**.

The GaN film was grown on LT-AlN substrates under NGCs with HVPE. Figure 3 shows photographic and optical microscopic images for samples A and B. When the LT-AlN was not annealed before the GaN growth, in sample A, the GaN layer with a thickness of 20 μm demonstrated a gray color, as shown in Figure 3A, and mesh-like sub-surface cracks appeared in the DCIM in Figure 3B. Furthermore, as shown in Figure 3C, the GaN film and its substrate cracked into pieces when the thickness reached ~100 μm. However, as shown in Figures 3D,F, for sample B with LT-AlN pre-annealed at 1070°C, the GaN film with the same thickness as sample A brightens and becomes transparent. In the more detailed image shown in Figure 3E, most of the GaN film showed smooth, stepwise formation with no cracks. This confirms that the majority of cracks in the GaN film were effectively suppressed by pre-annealing of LT-AlN at high temperatures, although local micro-cracks continued to exist, as shown in the insert to Figure 3E. Thus, pre-annealing LT-AlN is a main factor for the improvement in the quality of the crystal for the GaN film, which is in accordance with previous reports (Liu et al., 2009; Yoshizawa et al., 2018).

**Figure 4:**
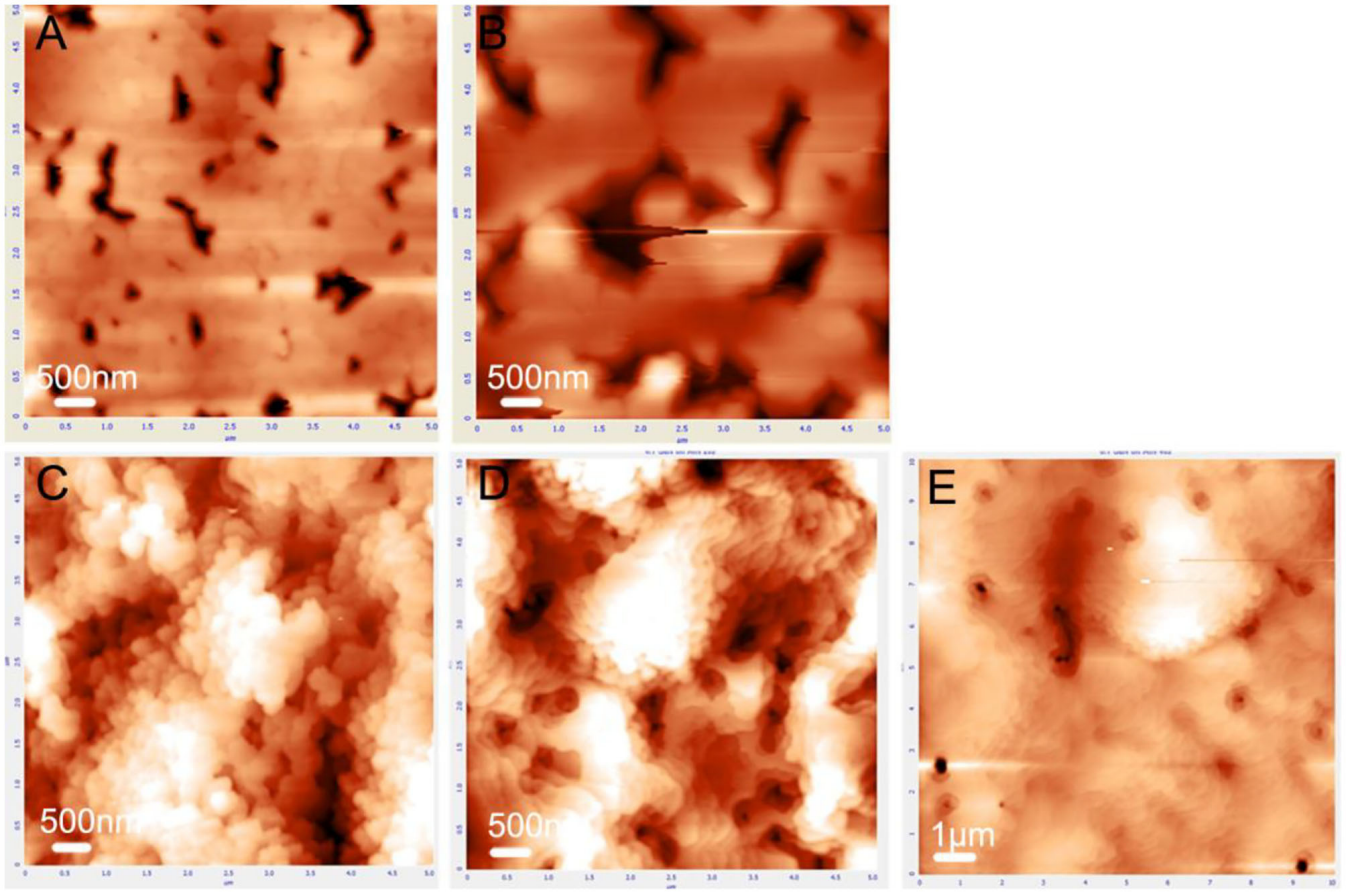
AFM images of GaN nucleation layer of sample A **(A,B)** and sample B **(C–E)**. Nucleation layer growth times were set to 10 s for **(A,C)**, 20 s for **(B,D)**, and 40 s for **(E)**.

It has been reported that the crystal properties of the GaN layer were mainly determined by the properties of the underlying AlN buffer layer through an influence on the nucleation of GaN (Yoshizawa et al., 2018). To study the underlying growth mechanism, the evolution of the surface morphology of GaN nucleation advanced with time was observed (Figure 4). First, AFM images of the GaN nucleation layer grown by HVPE for 10 and 20 s on top of LT-AlN without annealing are exhibited in Figures 4A,B, respectively. Moreover, Figures 4C–E shows a time series of AFM images of the GaN nucleation layer grown by HVPE for 10, 20, and 40 s on LT-AlN with annealing pre-treatment, respectively. From these images, it is clear that the behavior of the GaN nucleation on the pre-annealed LT-AlN was significantly different from that appearing under no annealing treatment. For the no-annealing sample, the GaN nucleation layer began to merge at 10 s, and many deep pits formed as a result, as shown in Figure 4A. These pits became larger with time, as shown in Figure 4B. These large pits then became the source of cracks and further expanded as the GaN film grew, as shown in Figures 3A–C. However, for the annealed sample, nucleation formed 3D GaN islands at 10 s, as shown in Figure 4C, and then these islands enlarged in a clear stepwise formation as shown in Figure 4D. Finally, the nucleation layer for GaN coalesced with smaller pits until 40 s, as shown in Figure 4E. This delayed coalescence behavior for the GaN nucleation layer blocked the propagation of dislocation and of cracks, which led to an improvement in the crystal quality of the GaN film grown on the annealed LT-AlN film (Shang et al., 2015).

### Growth of Crack-Free 2-Inch GaN Thick Wafers With High Crystal Quality Using CBL

**Figure 5:**
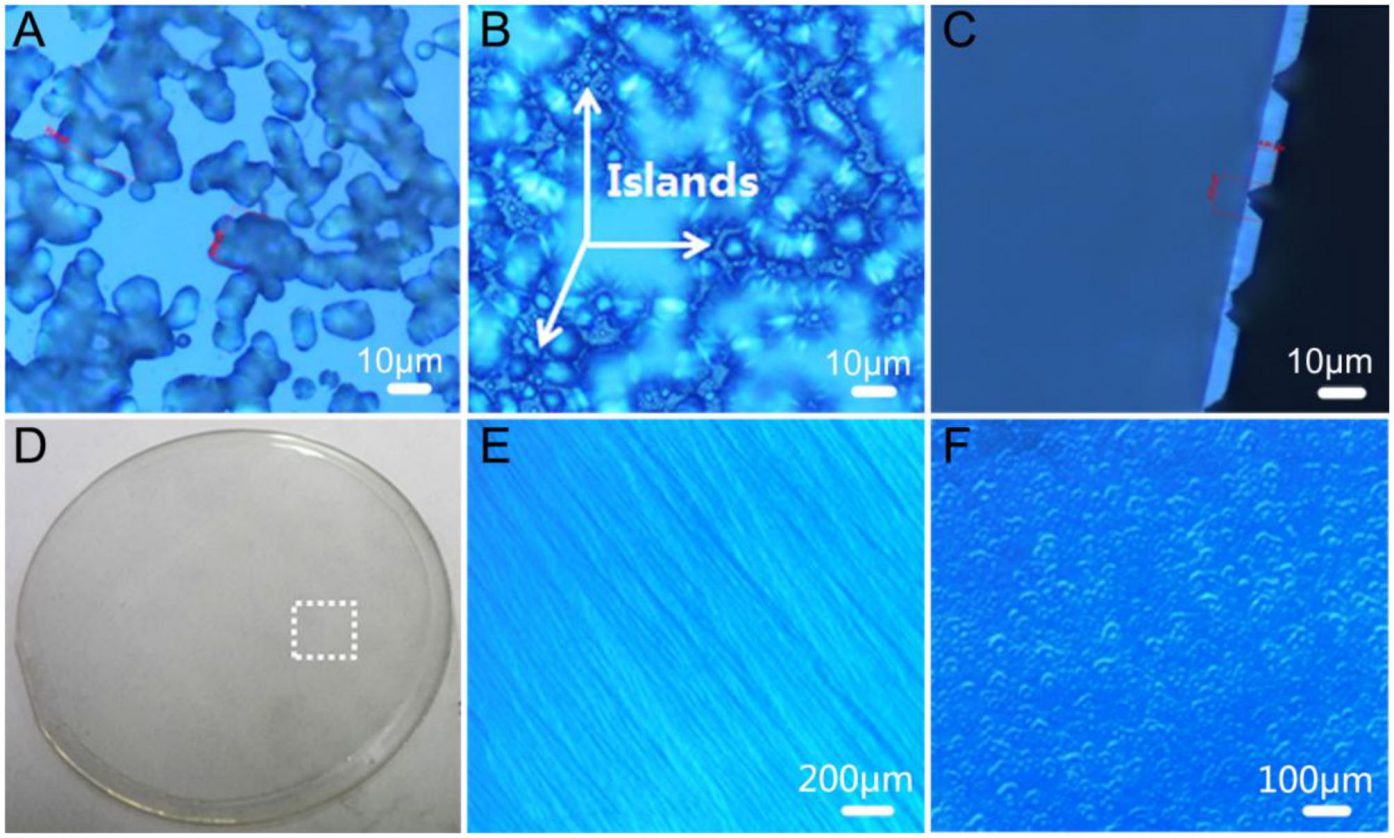
Optical images of the GaN 3D interlayer grown on LT-AlN with annealing pre-treatment focused on the surface **(A)** and near the interface between the GaN epitaxial layer and the LT-AlN-covered sapphire substrate **(B)**. **(C)** Microscopic mage of the cross-section of **(A)**. **(D)** Photographic image of sample D. Microscope images of the dotted white rectangle in **(D)** when focused on surface morphology **(E)** and near the interface between GaN epitaxial layer and the LT-AlN-covered sapphire substrate **(F)**.

According to the above analyses, the crystal properties of GaN film grown under NGCs were greatly improved by the use of pre-annealed LT-AlN. However, as shown in Figure 3E, sub-surface micro cracks also appeared that would detract from the ability to obtain a thick GaN film and a free-standing GaN substrate. It has been reported that the lateral overgrowth of GaN could be passivated under rich ambient H_2_ gas and change the GaN nucleus into 3D islands for the use of both MOCVD (Tadatomo et al., 1999) and the HVPE technique (André et al., 2012; Lekhal et al., 2016). This results in high single-crystal quality for GaN film, due to dislocation bending and termination on the inclined planes (Hiramatsu et al., 2000; Imade et al., 2011). Optical microscopic images for sample C are shown in Figures 5A–C. Here, a 3D GaN interlayer grown under an excess large flow of H_2_ gas was introduced on top of the annealed LT-AlN, and the growth process was immediately terminated when the GaN 3D interlayer growth was accomplished. In relation to the picture focused on the surface of the GaN 3D interlayer, as shown in Figure 5A, multiple surface pits larger than 10 μm were obtained by adding an additional high flow rate for the H_2_ gas. Moreover, as shown in Figure 5B, the image showing near interface between the GaN 3D interlayer and the LT-AlN-covered sapphire substrate demonstrates that a large number of islands formed among the pits. The depth of the deepest pits was ~4.8 μm, as shown in the cross-section in Figure 5C.

A 2-inch GaN wafer with a thickness of 250 μm (sample D) was obtained by adding a coalescence GaN layer and a normal GaN layer above the sample C. In the photographic image presented in Figure 5D, it can be seen that sample D is extremely bright and transparent, and the morphology and crystal quality of the GaN film are greatly improved by the perfect stepwise formation seen in Figure 5E. From the XRD rocking curve, measured with a slit width of 0.5 mm, the FWHMs of (002) and (102) for the XRD in sample D decreased from 245 and 412 to 123 and 151 arcsec, respectively, compared to sample B, grown under NGCs. Focusing on the interface between the GaN epitaxial layer and the LT-AlN covered sapphire substrate, shown in Figure 5F, many dense GaN 3D islands can be seen. It can be speculated that the shape of GaN islands could be kept unchanged for longer, and the coalescence could be delayed by adding a mass flow of H_2_ gas. In addition, the formation of GaN 3D islands could enhance the crystal quality and relax the stress. On the basis of these analyses, therefore, it can be concluded that combing the pre-annealing of the LT-AlN and adding the extra high flow rate of H_2_ gas could effectively improve the properties of the epitaxial GaN film due to the increased size and crystalline quality of the AlN nucleus by annealing, along with the 3D GaN interlayer formed by adjusting the lateral growth by H_2_ gas.

### Fabrication of a 2-Inch Free-Standing GaN Wafer Using LLO

**Figure 6:**
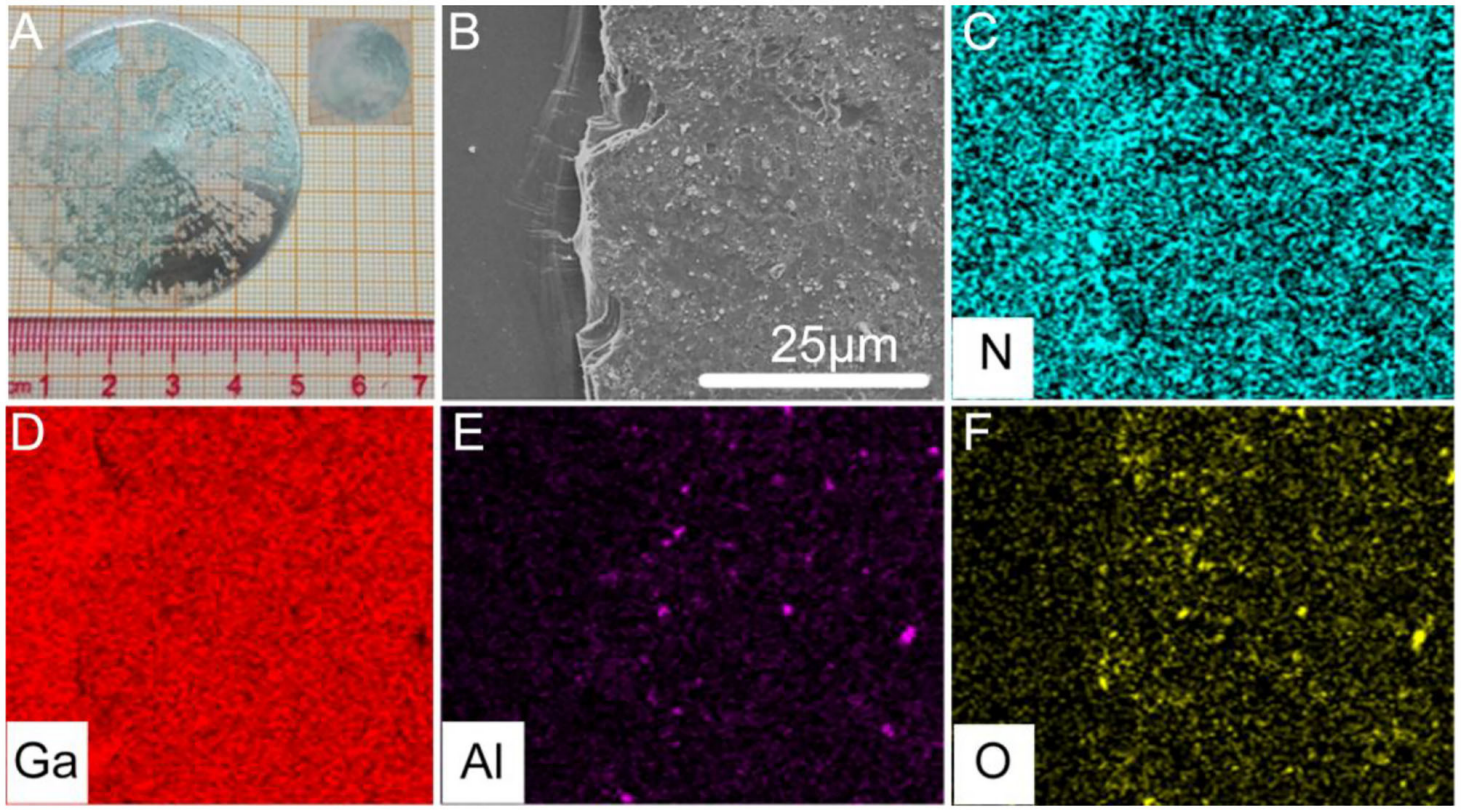
**(A)** Photographic image of 2-inch free-standing GaN wafer in Figure 5D after the sapphire substrate is removed with LLO. **(B)** SEM image for local area of insert of **(A)** after immersion in hot concentrated HCl at 50°C for 30 min. **(C–F)** EDS mapping analyses for the elements N, Ga, Al, and O in **(B)**, respectively.

The sapphire substrate of sample D, shown in Figure 5D, was removed with LLO, and a 2-inch free-standing GaN wafer was obtained. As seen in Figure 6A, it was found that the black parts existing on the N-face of the free-standing GaN substrate could not be dissolved in hot hydrochloric acid (HCl), as shown in the insert to Figure 6A. Figure 6B shows the SEM image for the local area of 2-inch free-standing GaN substrate after immersion in hot HCl at 50°C for 30 min, and EDS mapping analyses for the elements N, Ga, Al, and O are shown in Figures 6C–F, respectively. A much denser pattern of blue dots denoted as N elements are shown in Figure 6C, and red dots denoted as Ga elements are shown in Figure 6D confirm that the left part of the picture in Figure 6B is GaN film. Moreover, the much brighter and denser violet dots, representing Al, as shown in Figure 6E, and yellow dots, representing O, shown in Figure 6F illustrate that the bugle on the right part of the picture in Figure 6B is really a residual part of an oxidized AlN layer. Thus, it is speculated that part of the LT-AlN layer below the pits might be melted due to giant local heat instantaneously released from the decomposition of the GaN with the laser, which might primarily attack the pits formed within the 3D GaN layer, and the black parts existing on the N-face of a GaN free-standing substrate as the residual parts of the LT-AlN layer, which did not melt during the LLO process. This partly melted LT-AlN layer may act as a protecting buffer layer, blocking the shock-wave during LLO process (Safadi et al., 2005; Su et al., 2013).

## Conclusion

In summary, 2-inch crack-free free-standing GaN substrates with high crystal quality were obtained using the CBL and LLO techniques. Large AlN nuclei with an orderly orientation were obtained by annealing at high temperature, which led to the improvement of the crystal quality of GaN nuclei grown on it. Moreover, the addition of an excessive large flow of H_2_ gas during HVPE growth further increased the size of the GaN nucleus and delayed the coalescent growth as well. Therefore, the combination of LT-AlN and a 3D GaN interlayer plays a critical role in improving the properties of GaN film grown on top of it. In the meantime, LT-AlN buffer layer may have protected GaN film from rupture by partial melting during LLO processing, and thus a crack-free 2-inch free-standing GaN substrate was obtained. This suggests that the CBL method might be useful for the fabrication of free-standing GaN substrates and GaN-based devices.

## Data Availability Statement

The original contributions presented in the study are included in the article/supplementary material, further inquiries can be directed to the corresponding authors.

## Author Contributions

All authors listed have made a substantial, direct and intellectual contribution to the work, and approved it for publication.

## Conflict of Interest

The authors declare that the research was conducted in the absence of any commercial or financial relationships that could be construed as a potential conflict of interest.

## References

[B1] André Y. Trassoudaine A. Gil E. Lekhal K. Chelda-Gourmala O. Castelluci D.. (2012). Demonstration of crystal-vapor equilibrium leading to growth blockade of GaN during selective area growth. J. Cryst. Growth 354, 135–141. 10.1016/j.jcrysgro.2012.05.026

[B2] Chai X. Z. Qu B. Y. Liu P. Jiao Y. C. Zhu Y. S. Fang X. Q.. (2018). Reduction of yellow and blue luminescence in Si-doped GaN by rapid thermal annealing. J. Opt. 47, 511–515. 10.1007/s12596-018-0473-y

[B3] Chen S. W. Li H. Lu T.-C. (2016). Improved performance of GaN based light emitting diodes with ex-situ sputtered AlN nucleation layers. AIP Adv. 6:045311. 10.1063/1.4947299

[B4] Chen Y. A. Kuo C. H. Wu J. P. Chang C. W. (2015). Interruption-free growth of 10 μm-thick GaN film prepared on sputtered AlN/PSS template by hydride vapor phase epitaxy. J. Cryst. Growth 426, 180–185. 10.1016/j.jcrysgro.2015.05.011

[B5] Fujito K. Kubo S. Nagaoka H. Mochizuki T. Namita H. Nagao S.. (2009). Bulk GaN crystals grown by HVPE. J. Cryst. Growth 311, 3011–3014. 10.1016/j.jcrysgro.2009.01.046

[B6] Han S. Yang S. Sheng K. (2018). High-Voltage and High-ION/IOFF Vertical GaN-on-GaN Schottky barrier diode with nitridation-based termination. IEEE Electron. Device Lett. 39, 572–575. 10.1109/LED.2018.2808684

[B7] Hiramatsu K. Nishiyama K. Onishi M. Mizutani H. Narukawa M. Motogaito A.. (2000). Fabrication and characterization of low defect density GaN using facet-controlled epitaxial lateral overgrowth (FA-CELO). J. Cryst. Growth 221, 316–326. 10.1016/S0022-0248(00)00707-7

[B8] Imade M. Hirabayashi Y. Miyoshi N. Yoshimura M. Kitaoka Y. Sasaki T.. (2011). Control of growth facets and dislocation propagation behavior in the Na-flux growth of GaN. Cryst. Growth Des. 11, 2346–2350. 10.1021/cg2000443

[B9] Lee H.-J. Ha J.-S. Yao T. Kim C. Hong S.-K. Chang J. H.. (2009). Microstructural analysis of void formation due to a NH_4_Cl layer for self-separation of GaN thick films. Cryst. Growth Des. 9, 2877–2880. 10.1021/cg900193k

[B10] Lekhal K. Bae S.-Y. Lee H.-J. Mitsunari T. Tamura A. Deki M.. (2016). Controlled morphology of regular GaN microrod arrays by selective area growth with HVPE. J. Cryst. Growth 447, 55–61. 10.1016/j.jcrysgro.2016.05.008

[B11] Li X. Wang Y. J. Hane K. Shi Z. Yan J. (2018). GaN-based integrated photonics chip with suspended LED and waveguide. Opt. Commun. 415, 43–47. 10.1016/j.optcom.2017.12.077

[B12] Liu B. Gao J. Wu K. M. Liu C. (2009). Preparation and rapid thermal annealing of AlN thin films grown by molecular beam epi-taxy. Solid State Commun. 149, 715–717. 10.1016/j.ssc.2009.02.008

[B13] Liu N. L. Cheng Y. T. Wu J. J. Li X. B. Yu T. J. Xiong H.. (2016). HVPE homoepitaxial growth of high quality bulk GaN using acid wet etching method and its mechanism analysis. J. Cryst. Growth 454, 59–63. 10.1016/j.jcrysgro.2016.08.038

[B14] Liu N. L. Wang Q. Zheng X. P. Li S. F. Dikme Y. Xiong H.. (2018). The influence of V/III ratio on GaN grown on patterned sapphire substrate with low temperature AlN buffer layer by hydride vapor phase epitaxy. J. Cryst. Growth 500, 85–90. 10.1016/j.jcrysgro.2018.07.014

[B15] Liu X. K. Liu Q. Li C. Wang J. F. Yu W. J. Xu K.. (2017). 1.2 kV GaN Schottky barrier diodes on free-standing GaN wafer using a CMOS-compatible contact material. Jpn. J. Appl. Phys. 56:026501. 10.7567/JJAP.56.026501

[B16] Okuno K. Oshio T. Shibata N. Honda Y. Yamaguchi M. Tanaka S.. (2013). Structural evolution of AlN buffer and crystal quality of GaN films on a- and c-sapphire grown by metal organic vapor phase epitaxy. Phys. Status Solidi C 10, 369–372. 10.1002/pssc.201200587

[B17] Paskova T. Darakchieva V. Paskov P. P. Monemar B. Bukowski M. Suski T.. (2006). Bending in HVPE GaN free-standing films: effects of laser lift-off, polishing and high-pressure annealing. Phys. Status Solidi C 3, 1475–1478. 10.1002/pssc.200565412

[B18] Safadi M. R. Thakur J. S. Auner G. W. (2005). Laser ablation of AlN films grown on sapphire substrate. J. Appl. Phys. 97:084901. 10.1063/1.1863420

[B19] Sandvik P. Mi K. Shahedipour F. McClintock R. Yasan A. Kung P.. (2001). AlxGa1-xN for solar-blind UV detectors. J. Cryst. Growth 231, 366–370. 10.1016/S0022-0248(01)01467-1

[B20] Shang L. Lu T. P. Zhai G. M. Jia Z. G. Zhang H. Ma S. F.. (2015). The evolution of a GaN/sapphire interface with different nucleation layer thickness during two step growth and its influence on the bulk GaN crystal quality. RSC Adv. 5, 51201–51207. 10.1039/C5RA08369A

[B21] Su X. J. Xu K. Xu Y. Ren G. Q. Zhang J. C. Wamg J. F.. (2013). Shock-induced brittle cracking in HVPE-GaN processed by laser lift-off techniques. J. Phys. D 46:205103. 10.1088/0022-3727/46/20/205103

[B22] Sumiya M. Toyomitsu N. Nakano Y. Wang J. Y. Harada Y. Sang L. W.. (2017). Deep-level defects related to the emissive pits in thick InGaN films on GaN template and bulk substrates. APL Mater. 5:016105. 10.1063/1.4974935

[B23] Tadatomo K. Ohuchi Y. Okagawa H. Itoh H. Miyake H. Hiramatsu K.. (1999). Hydrogen and nitrogen ambient effects on epitaxial lateral overgrowth (ELO) of GaN via metal organic vapor-phase epitaxy (MOVPE). MRS Int. J. Nitride Semicond. Res. 4:G3.1. 10.1557/S1092578300002325

[B24] Yoshizawa R. Miyake H. Hiramatsu K. (2018). Effect of thermal annealing on AlN films grown on sputtered AlN templates by metal organic vapor phase epitaxy. *Jpn. J*. *Appl*. Phys. 57:01AD05. 10.7567/JJAP.57.01AD05

[B25] Zhao D. G. Jiang D. S. Zhu J. J. Liu Z. S. Zhang S. M. Yang H.. (2007). The influence of V/III ratio in the initial growth stage on the properties of GaN epilayer deposited on low temperature AlN buffer layer. J. Cryst. Growth 303, 414–418. 10.1016/j.jcrysgro.2007.01.019

